# Knowledge, attitudes and practices of registered nurses regarding the spread of nosocomial infections and the impact of organizational support

**DOI:** 10.1186/2047-2994-4-S1-P60

**Published:** 2015-06-16

**Authors:** E Kamunge, T Cahill, G Zipp, R Parasher

**Affiliations:** 1 Bio/Chem, Essex County College, Newark, NJ, USA; 2 Graduate School of Medical Education, Seton Hall University, South Orange, NJ, USA

## Introduction

Nosocomial infections (NIs) are new localized or systemic infections that develop in patients receiving medical care in a hospital or other healthcare facilities. They are caused by pathogens often transmitted by indirect and direct contact. It has been documented in the literature that at the time of graduation from their professional education, healthcare professionals have sufficient knowledge to practice safe patient-care and to follow infection control guidelines. However, the evidence suggests otherwise since healthcare professionals, across all categories, are implicated in the transmission of NIs. With nurses having the most contacts with patients, understanding of their knowledge, attitudes and practice patterns with regard to the spread of NIs may provide one approach by which this health care issue would be addressed.

## Objectives

The study investigated whether differences exist between novice and experienced registered nurses' knowledge, attitudes and practices; and further explored the impact of organizational support with regards to the spread of nosocomial infections.

## Methods

This exploratory, cross-sectional and descriptive study was conducted using on-line survey responses from 352 registered nurses. Data was analyzed with descriptive and inferential non-parametric statistics.

## Results

The participants demonstrated high levels of knowledge regarding the spread of nosocomial infections, adherence to recommended guidelines of infection control practices, and positive attitudes. The results of correlation analysis indicated a significant positive correlation between organizational support and respondents' knowledge; and weak but significant positive correlations between organizational support and respondents’ attitudes and practices in respective categories.

## Conclusion

Registered nurses, both the novice and experienced, had good knowledge about the spread of NIs, practised safe patient-care protocols, and had positive attitudes. Additionally, results of data analysis suggest that organizational support plays pivotal role toward reducing the spread of NIs.

## Disclosure of interest

None declared.

